# A series of cutaneous manifestations in a patient with vacuoles, E1 enzyme, X-linked, autoinflammatory, somatic syndrome

**DOI:** 10.1177/2050313X251341512

**Published:** 2025-05-24

**Authors:** Anne-Sophie Groleau, Alex Derstenfeld, Steffany Poupart, Isabelle Marcil

**Affiliations:** 1 Division of Dermatology, Department of Medicine, Centre Hospitalier Universitaire de Montreal (CHUM), Université de Montréal, QC, Canada; 2 Division of Dermatology, Department of Medecine, Université de Montréal, QC, Canada

**Keywords:** VEXAS syndrome, Sweet-like syndromes, neutrophilic dermatosis, neutrophilic panniculitis

## Abstract

Vacuoles, E1 enzyme, X-linked, autoinflammatory, somatic is a rare, autoinflammatory disorder caused by a somatic mutation of ubiquitin-activating enzyme 1. Common systemic manifestations include fever, neutrophilic dermatoses, relapsing polychondritis, and pulmonary manifestations. Cutaneous manifestations are frequently the initial presentation and are a marker of systemic disease. Here, we present an unusual case of a 71-year-old man with multiple cutaneous manifestations of vacuoles, E1 enzyme, X-linked, autoinflammatory, and somatic syndrome occurring simultaneously.

## Report of a case

Vacuoles, E1 enzyme, X-linked, autoinflammatory, somatic (VEXAS) is a rare, autoinflammatory disorder. Here, we present a case of the multiple cutaneous manifestations of VEXAS syndrome occurring simultaneously in a patient.

A 71-year-old man, known for VEXAS, with an identified ubiquitin-activating enzyme 1 (UBA1) mutation, presented to a dermatology clinic for consultation. The patient was initially referred for asymptomatic erythematous papules located on the right forearm and palms. The patient was known for concomitant myelodysplastic syndrome, polychondritis, and polyarthritis associated with VEXAS for which he receives azacytidine and tocilizumab, respectively.

**Figure 1.**
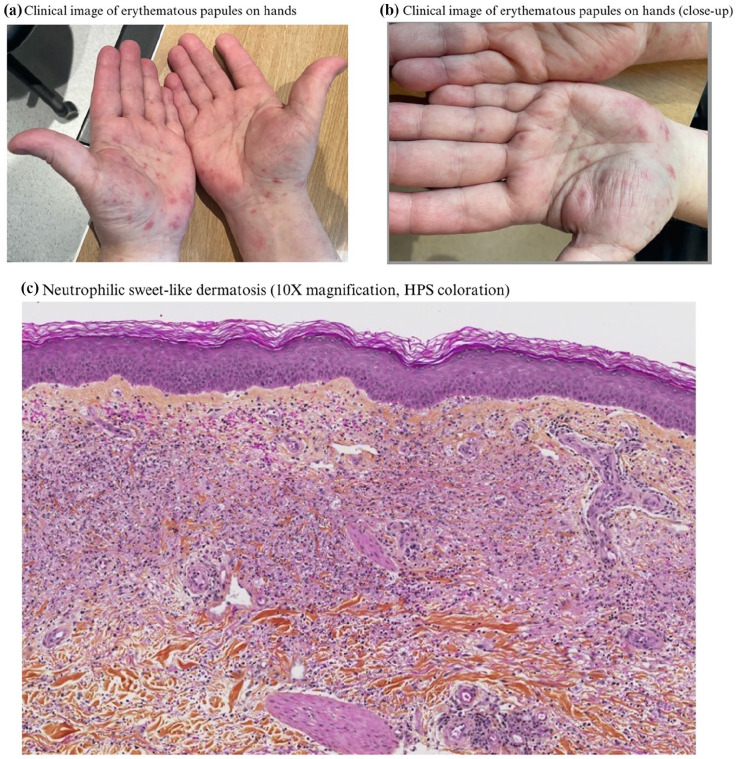
(a) Small, erythematous papules on the palms and right wrist. (b) Close-up of palms. (c) Diffuse infiltration of the papillary dermis with leucocytoclasia and histiocytic infiltrates. No evidence of vasculitis, fungal or mycobacterial infection or of leucemic infiltrate. Findings are compatible with a “sweet-like” neutrophilic dermatosis.

Upon presentation, the patient appeared well and was afebrile. Multiple small (2–3 mm) well-circumscribed erythematous papules without overlying epidermal changes were present on his palms (Figure 1(a)) as well as the ventral aspect of the right forearm. A 4 mm punch biopsy was performed, and the patient was treated with betamethasone dipropionate 0.05% cream twice daily. On histopathology, findings were compatible with a “sweet-like” neutrophilic dermatosis (Figure 1(c)). At follow-up 4 weeks later, there was complete resolution of the lesions.

**Figure 2.**
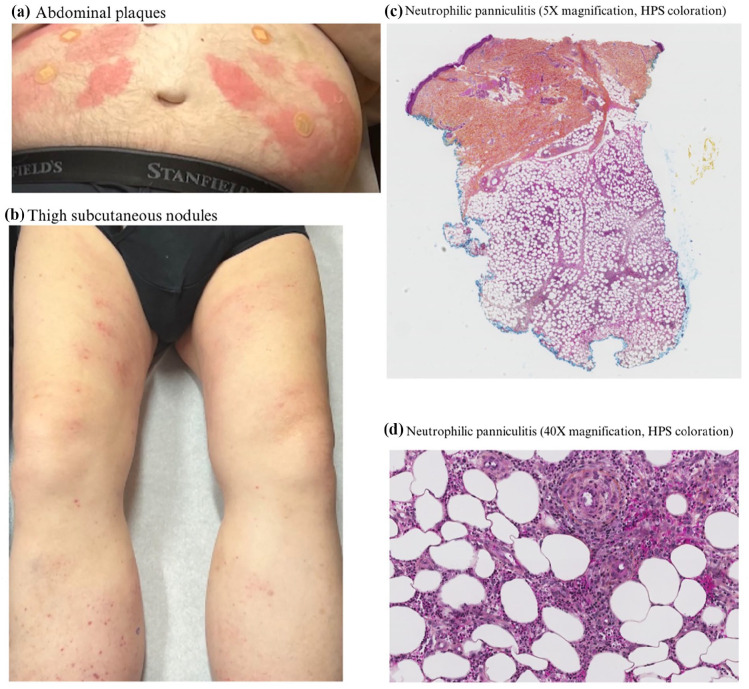
(a) Well-circumscribed erythematous round plaques on the abdomen with biopsy sites. (b) Firm subcutaneous nodules on the thighs with overlying petechiae and non-palpable purpura. (c) Lobular and neutrophilic panniculitis compatible with a neutrophilic panniculitis associated with VEXAS syndrome. (d) Lobular and neutrophilic panniculitis magnified to 40×. VEXAS: vacuoles, E1 enzyme, X-linked, autoinflammatory, somatic.

A few weeks later, the patient presented to the clinic anew with lesions constituting multiple simultaneously occurring morphologies. On the forearms, the patient presented with diffuse erythematous infiltrated papules. On the abdomen were additional well-circumscribed erythematous round plaques (Figure 2(a)). Lastly, on the lower extremities, the patient manifested firm subcutaneous nodules (Figure 2(b)), some of which had overlying petechiae and non-palpable purpura. Two 4 mm punch biopsies were taken from the abdomen and right thigh, respectively.

Histopathology of the abdomen demonstrated a neutrophilic infiltrate in the superficial dermis with leukocytoclasia, in favor of a “sweet-like” neutrophilic dermatosis compatible with VEXAS. These histological changes were superimposable to those seen previously at the right forearm. The biopsy of the right thigh demonstrated lobular neutrophilic panniculitis, also compatible with VEXAS (Figure 2(c) and (d)). On follow-up, the patient had complete resolution of his lesions.

## Discussion

VEXAS is a rare autoinflammatory disease that typically presents in men 50 years and older with systemic inflammation. VEXAS is caused by an acquired somatic mutation of UBA1 in cells of myeloid and erythroid origin.^1,2^ VEXAS’ protean manifestations most commonly include fever, neutrophilic dermatoses, relapsing polychondritis, arthritis, pulmonary manifestations but may affect any organ system.^2^ Concomitant myelodysplastic syndrome has been reported in up to 24% of patients, and, in those cases, cutaneous lesions have been demonstrated to be composed of infiltrates from the myeloid clone.^3^ Cutaneous involvement is frequent and may constitute the initial presentation; cutaneous involvement is considered an important marker of both disease activity and systemic inflammation.^2,4^ Described dermatologic manifestations include small and medium-vessel vasculitis, neutrophilic dermatoses, urticaria-like lesions, periorbital edema, and chondritis.^2^ Commonly reported skin histological findings include leukocytoclastic vasculitis and neutrophilic dermatoses.^4,5^ Interestingly, the leucine variant of the UBA1 mutation has been associated with neutrophilic dermatoses Sweet-like syndrome phenotype.^6^

Our case constitutes the first case demonstrating the simultaneously occurring multiple cutaneous manifestations of VEXAS, including the seldom described neutrophilic panniculitis. What is more, said lesions occurred despite concomitant treatment with azacitidine. Management of such cases remains challenging as there is a paucity of data regarding effective therapeutic strategies.^4^ High-dose corticosteroids are often needed to address the immediate inflammatory manifestations.^2,7^ Steroid-sparing agents include JAK inhibitors, azacytidine and cyclosporin.^2,4^ Failing the aforementioned treatments, allogenic hematopoietic stem cell transplant has demonstrated promise as a potentially curative therapy.^2,4^
